# Role of the cofactor CTIP2 (COUP-TF Interacting Protein 2) in the transcriptional repression of HTLV-1 (Human T-lymphotropic Virus 1)

**DOI:** 10.1186/1742-4690-8-S1-A130

**Published:** 2011-06-06

**Authors:** Gwenaelle Robette, Benoit Van Driessche, Allan Guiguen, Arsène Burny, Olivier Rohr, Carine Van Lint

**Affiliations:** 1 Laboratory of Molecular Virology, Institut de Biologie et de Médecine Moléculaires (IBMM), Université Libre de Bruxelles (ULB), Gosselies, 6041, Belgium; 2 Institut Universitaire de Technologie Louis Pasteur de Schiltigheim, University of Strasbourg, Schiltigheim, 67300, France

## Background

Following entry and reverse transcription, HTLV-1 integrates into the host cell genome. The viral promoter activity is directly governed by its chromatin environment. Epigenetic modifications, such as DNA methylation and histone deacetylation, are crucial for transcriptional silencing of the virus. We have previously reported that the cofactor CTIP2 recruits a multienzymatic chromatin-modifying complex, including histone deacetylases and a methyltransferase to the HIV-1 promoter, thereby establishing a heterochromatic environment at the promoter. CTIP2 is recruited to the HIV-1 promoter via its association with the transcription factor Sp1. Here, we investigated the potential role of CTIP2 in transcriptional regulation of the HTLV-1 promoter.

## Methods

Electrophoretic mobility shift assays (EMSAs), transient transfection and chromatin immunoprecipitation (ChIP) assays were performed.

## Results

Analysis of the nucleotide sequence of the HTLV-1 promoter (LTRHTLV-1) revealed two Sp1 binding sites within the R region. We demonstrated that Sp1 and Sp3 bind in vitro to these sites by EMSAs. We showed, by cotransfection assays in epithelial HEK293T cells and T-lymphoid Jurkat cells, that the cofactor CTIP2 inhibited Tax-mediated transactivation of the HTLV-1 promoter. We demonstrated by ChIP assays the in vivo recruitment of CTIP2 to the LTRHTLV-1 in a latently HTLV-1 infected cell line (TLom1). Conversely, CTIP2 was absent from the proviral genome in a HTLV-1 productive cell line (SLB1). The identification of the multienzymatic complex recruited by CTIP2 to the viral promoter under latent conditions is currently under investigation.

## Conclusion

Our results demonstrate a repressive role of CTIP2 on LTRHTLV-1 transcriptional activity, suggesting a potential effect of this cofactor in HTLV-1 latency.

